# Supplementary material for: Impact of High-Pressure Homogenization Parameters on Physicochemical Characteristics, Bioactive Compounds Content, and Antioxidant Capacity of Blackcurrant Juice
Source: Molecules. 2021 Mar 23;26(6):1802. doi: 10.3390/molecules26061802 (PMC8004621; doi:10.3390/molecules26061802)
Supplement: Supplementary file 1 [file molecules-26-01802-s001.pdf]

molecules

MDPI

Supplementary materials

Impact of High-Pressure Homogenization Parameters on Physicochemical Characteristics, Bioactive Compounds Content and Antioxidant Capacity of Blackcurrant Juice

Bartosz Kruszewski [1,*], Katarzyna Zawada [2] and Piotr Karpiński [3]

[1] Department of Food Technology and Assessment, Institute of Food Sciences, Warsaw University of Life Sciences - SGGW, Nowoursynowska 159 C, 02-776 Warsaw, Poland
[2] Department of Physical Chemistry, Faculty of Pharmacy with the Laboratory Medicine Division, Medical University of Warsaw, Banacha 1, 02-097 Warsaw, Poland; kzawada@wum.edu.pl
[3] Faculty of Computer Science and Food Science, Lomza State University of Applied Sciences (PWSIiP), Akademicka 14, 18-400 Łomża, Poland. pkarpinski@pwsip.edu.pl
* Correspondence: bartosz_kruszewski@sggw.edu.pl; Tel.: +48 22 593 75 20

Table S1. L*, a*, b* colour values of cloudy blackcurrant juice samples treated with high-pressure homogenization or pasteurization.

Treatment			L*	a*	b*
Raw Juice			11.49 ± 0.01 ^{a}	43.36 ± 0.34 ^{j}	19.90 ± 0.17 ^{a}
Pasteurization 90 °C, 1 min			13.28 ± 0.04 ^{n}	40.91 ± 0.03 ^{ab}	22.90 ± 0.04 ^{i}
Homogenization					
Temp. °C	Pressure MPa	Number of Passes			
4	50	1	11.64 ± 0.02 ^{b}	43.06 ± 0.03 ^{i}	20.26 ± 0.07 ^{b}
		3	12.03 ± 0.02 ^{ef}	42.26 ± 0.02 ^{fg}	20.54 ± 0.04 ^{cde}
		5	12.44 ± 0.06 ^{i}	41.88 ± 0.04 ^{de}	20.20 ± 0.05 ^{b}
	150	1	11.74 ± 0.01 ^{c}	42.72 ± 0.07 ^{h}	20.45 ± 0.07 ^{c}
		3	12.06 ± 0.03 ^{f}	42.23 ± 0.02 ^{fg}	20.55 ± 0.03 ^{cde}
		5	12.32 ± 0.02 ^{h}	42.33 ± 0.03 ^{g}	20.48 ± 0.04 ^{cd}
	220	1	11.74 ± 0.03 ^{c}	42.94 ± 0.04 ^{hi}	20.52 ± 0.07 ^{cde}
		3	12.18 ± 0.05 ^{g}	42.34 ± 0.01 ^{g}	20.66 ± 0.01 ^{e}
		5	12.56 ± 0.04 ^{j}	41.64 ± 0.04 ^{c}	21.03 ± 0.03 ^{f}
20	50	1	12.43 ± 0.02 ^{i}	42.08 ± 0.03 ^{ef}	20.54 ± 0.03 ^{cde}
		3	12.80 ± 0.08 ^{k}	41.69 ± 0.05 ^{cd}	21.05 ± 0.06 ^{f}
		5	13.06 ± 0.06 ^{lm}	41.68 ± 0.01 ^{cd}	21.32 ± 0.07 ^{g}
	150	1	11.86 ± 0.03 ^{d}	42.83 ± 0.05 ^{hi}	20.60 ± 0.05 ^{cde}
		3	11.93 ± 0.02 ^{de}	42.30 ± 0.02 ^{fg}	20.56 ± 0.07 ^{cde}
		5	12.53 ± 0.01 ^{ij}	41.6 ± 0.03 ^{e}	20.62 ± 0.05 ^{de}
	220	1	13.15 ± 0.07 ^{m}	41.50 ± 0.06 ^{c}	21.28 ± 0.06 ^{g}
		3	13.15 ± 0.05 ^{m}	41.14 ± 0.04 ^{b}	21.32 ± 0.03 ^{g}
		5	13.03 ± 0.01 ^{l}	40.90 ± 0.06 ^{a}	21.53 ± 0.05 ^{h}

Different letters (a–l) in columns mean statistically significant differences at the level of $p < 0.05$.
